# Accumulated ambient air pollution and colon cancer incidence in Thailand

**DOI:** 10.1038/s41598-020-74669-7

**Published:** 2020-10-20

**Authors:** Kriangsak Jenwitheesuk, Udomlack Peansukwech, Kamonwan Jenwitheesuk

**Affiliations:** 1 General Surgery Unit, Department of Surgery, Faculty of Medicine, Khon Kaen University, Khon Kaen, Thailand; 2 CKDnet, Khon Kaen University, Khon Kaen, Thailand; 3 Plastic and Reconstructive Unit, Department of Surgery, Faculty of Medicine, Khon Kaen University, Khon Kaen, Thailand

**Keywords:** Cancer, Environmental sciences, Natural hazards, Gastroenterology

## Abstract

This research examined the relationship between colon cancer risks and pollution in various areas of Thailand, using satellites to gather quantities of aerosols in the atmosphere. Bayesian hierarchical spatio-temporal model and the Poisson log-linear model were used to examine the incidence rates of colon cancer standardized by national references; from the database of the National Health Security Office, Ministry of Public Health of Thailand and NASA’s database from aerosol diagnostics model. Modern-Era Retrospective Analysis for Research and Applications, Version 2 (MERRA-2) was used to explore disease-gender-specific spatio-temporal patterns of colon cancer incidences and accumulated air pollution-related cancers in Thailand between 2010 and 2016. A total of 59,605 patients were selected for the study. Due to concerns regarding statistical reliability between aerosol diagnostics model and colon cancer incidences, the posterior probabilities of risk appeared the most in dust PM_2.5_. It could be interpreted as relative risk in every increase of 10 μg/m^3^ in black carbon, organic carbon, and dust-PM_2.5_ levels were associated respectively with an increase of 4%, 4%, and 15% in the risks of colon cancer. A significant increase in the incidence of colon cancer with accumulated ambient air quality raised concerns regarding the prevention of air pollution. This study utilized data based on the incidences of colon cancer; the country’s database and linked cancer data to pollution. According to the database from NASA’s technology, this research has never been conducted in Thailand.

## Introduction

Nowadays cancer has become a common illness in our society. The incidence rate of cancer surveyed in 2012 was 14.1 million cases, 8.2 million of which were dead and 32.6 millions had only 5 years to survive. The majority of the cancer patients are poor people living in developing countries^1^. It has been found that cancer in developing countries is proportioned up to 57%, and nasopharynx cancer is three times as high^2^. The diet and lifestyle of the patients are also major contributing factors that influenced the cancer^3–5^. Environmental factors related to pollution, whether it is a result of toxic industrial waste or agricultural chemicals contaminate the environment also add to the growth of cancer^6^. Research in Taiwan discovered that those who drank groundwater with arsenic contamination for over 50 years, represented the majority for the fatalities for colon cancer, was higher than normal^7^. In the United States, arsenic, asbestos, radon, chlorine and agricultural chemicals, including hazardous waste increased the risk of cancer. Arsenic increases the risk of liver cancer, bladder cancer, and kidney cancer. The products from chlorine used to clean the tap water may increase the risk of rectal cancer and bladder cancer^8^. In Denmark, it found that those who drink water with nitrate contamination at the level of 9.3 mg/L were vulnerable to large bowel cancer, with an increase of percentage in comparison to those with less contact (less than 1.3 mg/L)^9^.

Air pollution in Thailand is one of the serious health issues. Overall ambient air quality from 63 automatic air quality monitoring stations located in 33 provinces revealed number of days in 2018 which the 24-h average exceeded the standard^10^. Many parts of the country have reported witnessing of an immense levels of environmental degradation. A popular tourist destination, Chiang Mai, in the northern region was hit by unprecedented levels of air pollution as intensifying forest fires sent PM_2.5_ levels up to 12 times above safe levels^11^. In 2019, the government was forced to close more than 400 schools in Bangkok due to toxic smog^12^. According to the International Agency for Research on Cancer report, Thailand had approximately 114,199 deaths related to cancer in 2018 and colorectal cancer (CRC) was the third leading cause in both sex^13^. This information reveals that Thailand has high risk of cancer fatalities. In-depth studies reveal the frequency of cancer in each organ which having varied risk factors and causes. This high cancer prevalence, therefore, may not be explained solely by behavioral risks^13^. Some researchers have reported that ambient air pollution in Thailand may increase the risk for lung cancer^14^.

Despite a possible link between the cancer and air pollution, there is a need for more evidence in terms of pathophysiology. Is it possible to utilize science and technology? Modern satellites technology was used to explore and detect the amount of aerosol chemical composition in source regions. It displayed the world's highest amount of aerosol chemical composition in 2006^15^. If the information on medical and geographic science can be linked together, this may provide a substantial amount of new knowledge.

At present medical advancements worldwide have a better management system. If most physicians have an understanding on the causes of the diseases, especially the risks of cancer in each location, this would help in providing suitable and effective prevention. The ability to link various pollution with the modern satellite technology system may allow the search process for the cancer’s risks to be more complete. If we can prevent these risk factors, it will help the quality of health amongst the world population. In addition, it may help to save every country’s resources. This present study, therefore, examined the relation between colon cancer and the associated risks and accumulated pollution in various areas of Thailand, using the satellites to explore the amount of toxic substances especially aerosols in ambient air.

## Material and method (Fig. 1)

**Figure 1:**
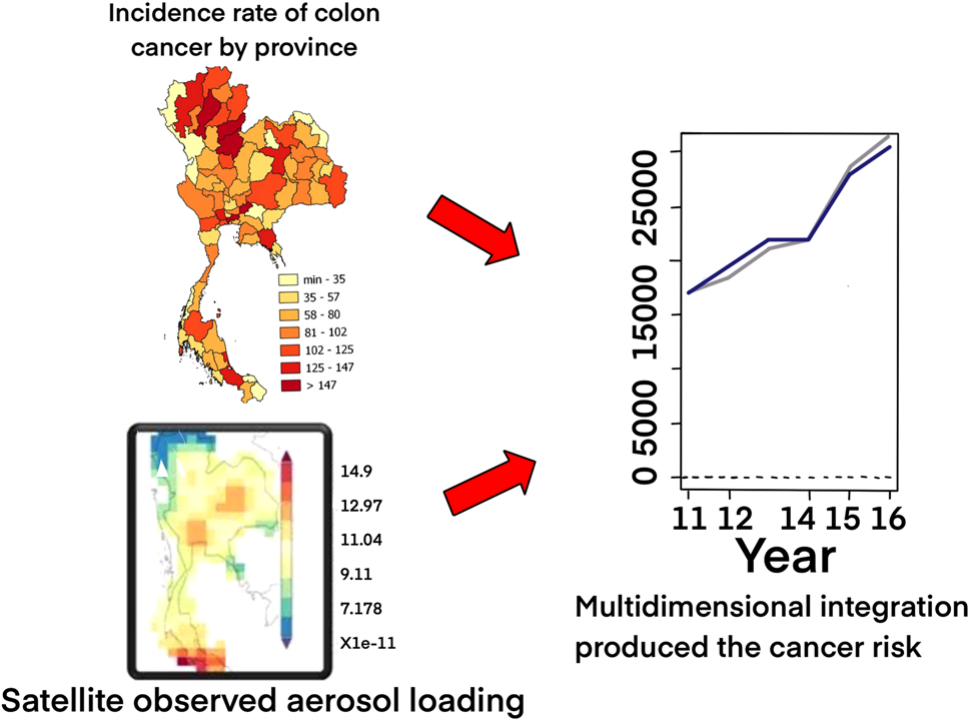
An investigation on spatial and temporal associations for the relative risk of accumulated air quality and incidence rates of colon cancer. (The maps were generated by QGIS software: a free and open source geographic information system, https://www.qgis.org/en/site/).

In this present study, the accumulated pollution was retrieved between 2010 and 2016, and the data of patients were collected in 2017 in order to examine the association between an exposure to the air pollution from the year 2010 to 2016 and the onset of colon cancer one year later in 2017.

We investigated spatial and temporal associations for the relative risk of air quality and incidence rates of colon cancer in Thailand between 2010 and 2016. The objective was to present the two different perspectives, the application of knowledge, and methodologies. The newly proposed spatial flexible parametric relative accumulated polluted-colon cancer risk model was extended to the spatio-temporal context.

### Ethical considerations

The study was reviewed and approved by the Khon Kaen University Ethics Committee for Human Research (HE631234). All methods were performed in accordance with the relevant guidelines and regulations. Data were obtained from two public domains which were opened for the public to use under noncommercial purposes. None of the variables or data used in this study allowed the identification of individuals. Confidentiality in this study was considered together with the privacy consideration, where relevant. The obligation to protect and promote the non-disclosure of information imparted in a relationship of trust lies at the core of the concept of confidentiality.

### Data collection

**Figure 2:**
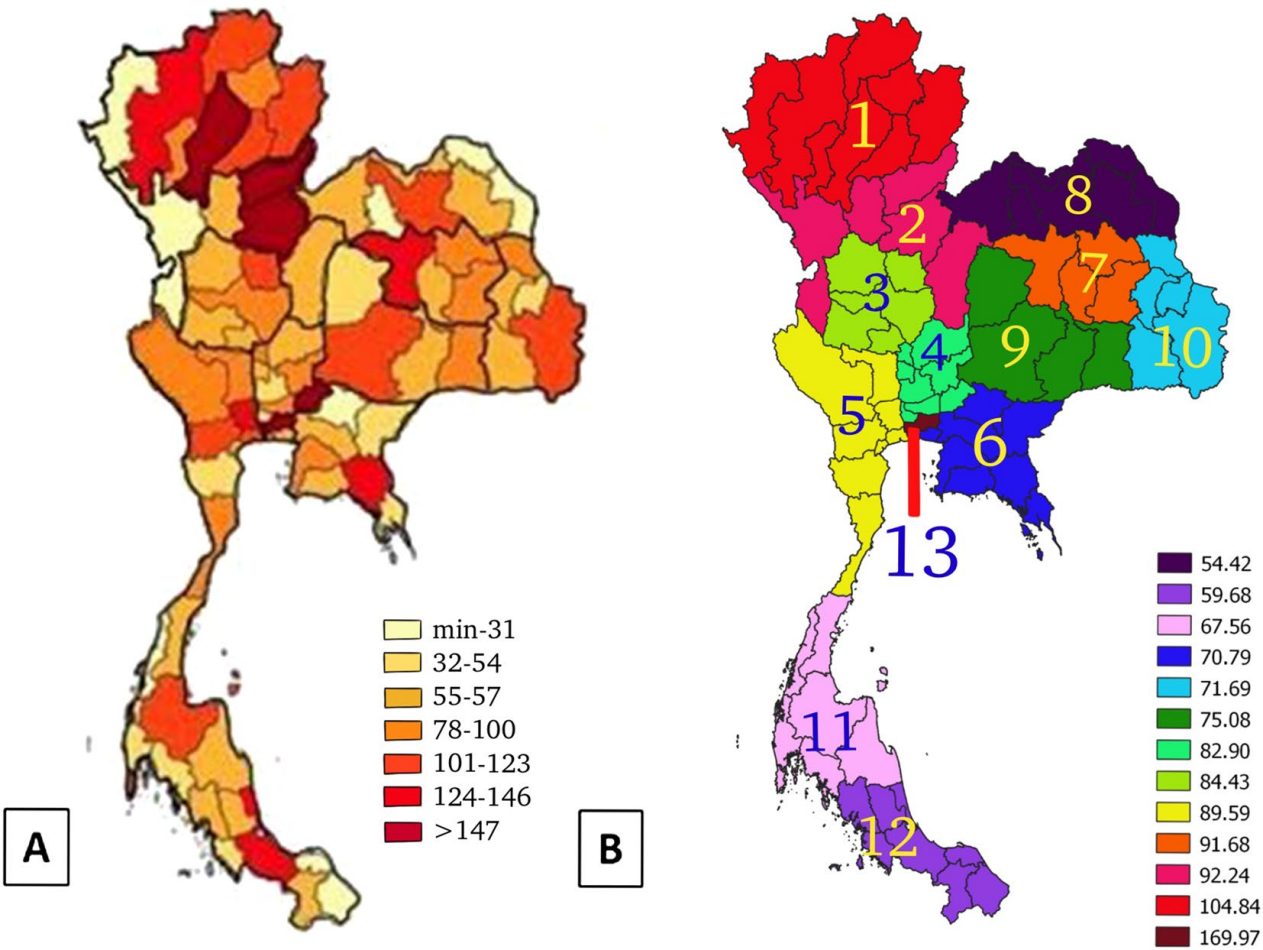
Map (**A**) represents the incidence rates (per 100,000 population) of colon cancer in each province and map (**B**) represents the 13 health regions incidence rates (per 100,000 population) of colon cancer. (The maps were generated by QGIS software: a free and open source geographic information system, https://www.qgis.org/en/site/).

**Figure 3:**
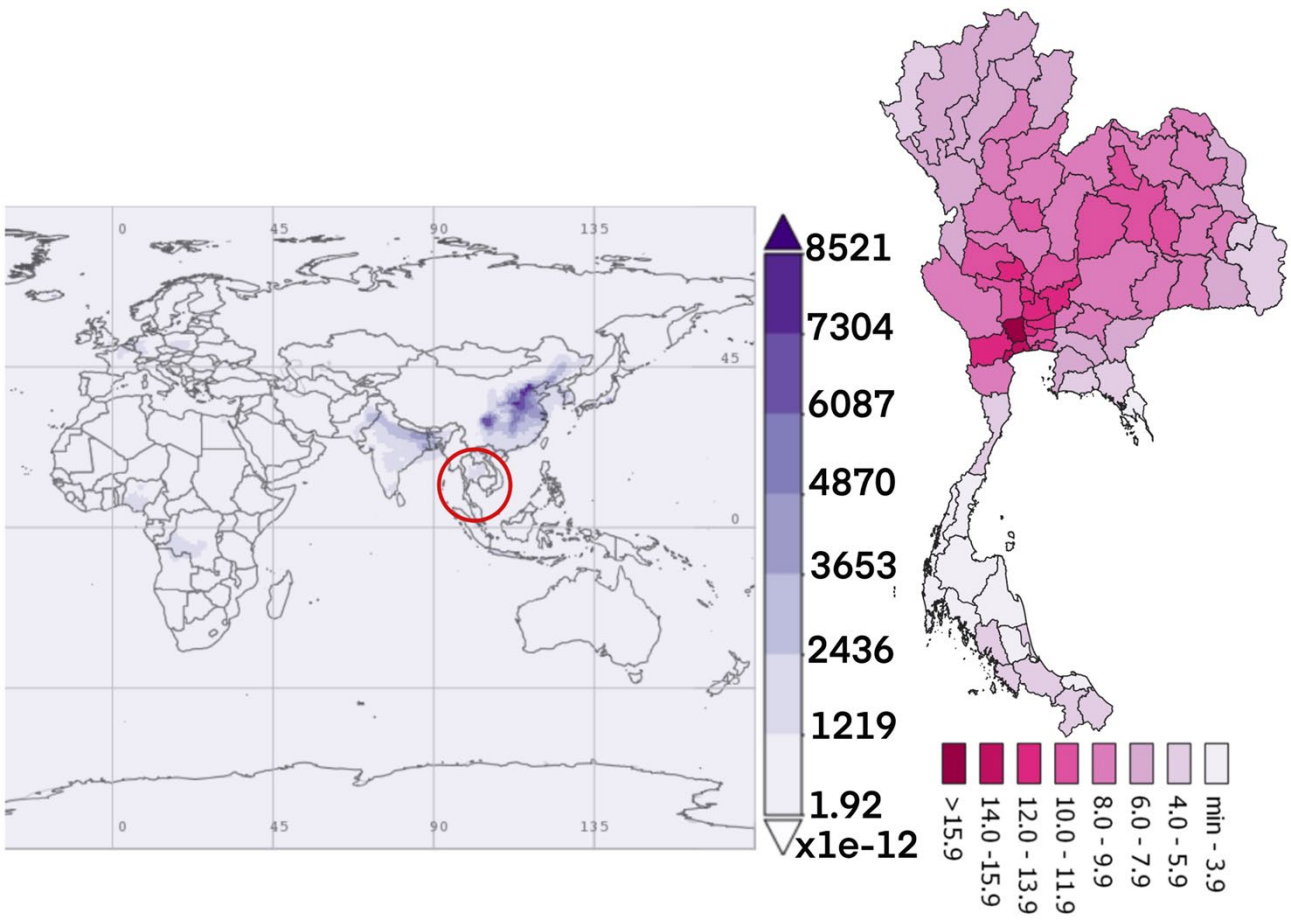
The average time of black carbon concentration monthly over 2010–2016. (The world map was generated by Giovanni Version 4.34: a free and open source earth data, https://giovanni.gsfc.nasa.gov/giovanni/) and accumulated dose between 2010 and 2016 in Thailand (Thailand map was generated by QGIS software: a free and open source geographic information system, https://www.qgis.org/en/site/).

**Figure 4:**
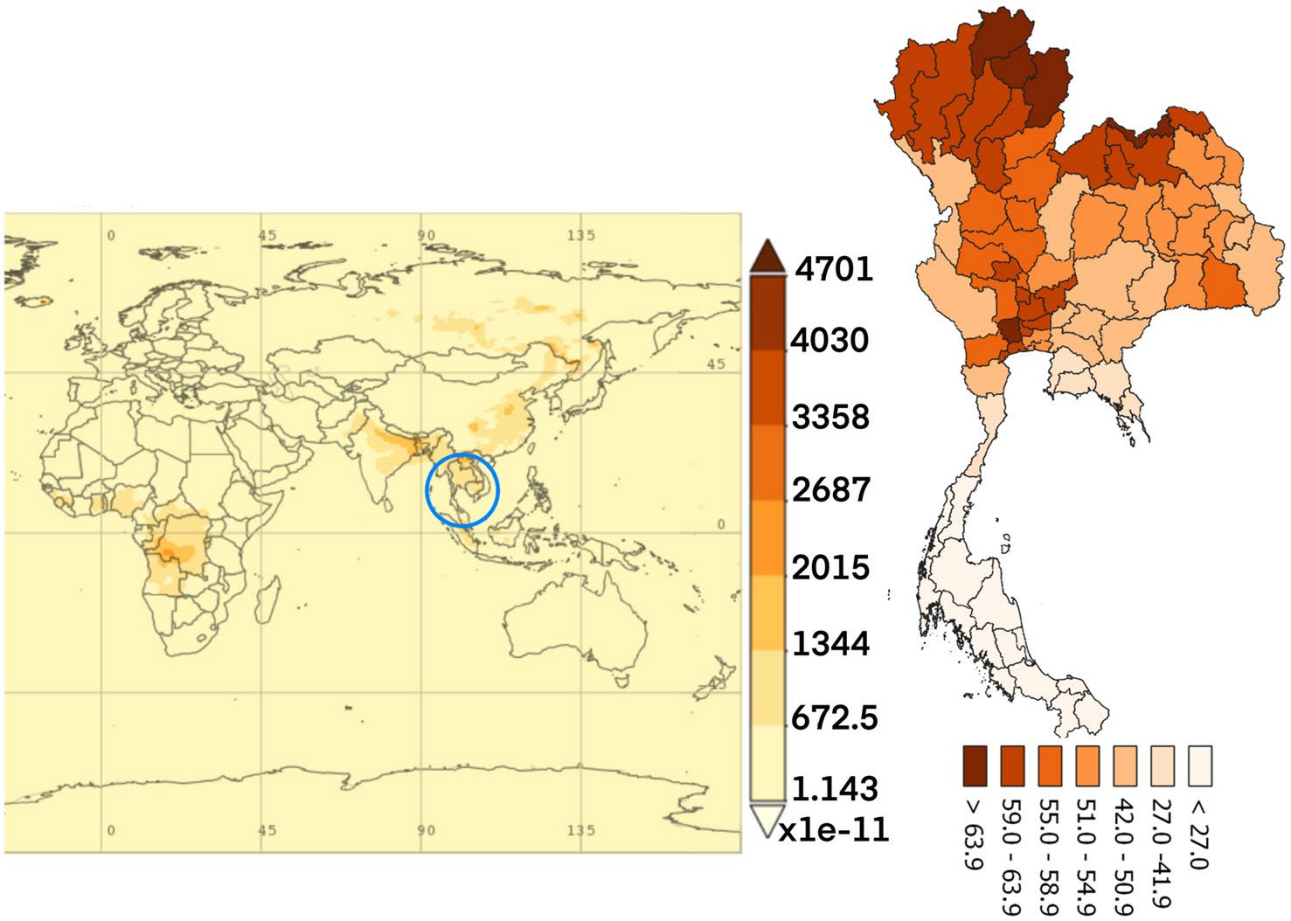
The average time of organic carbon concentration monthly over 2010–2016. (The world map was generated by Giovanni Version 4.34: a free and open source earth data, https://giovanni.gsfc.nasa.gov/giovanni/) and accumulated dose between 2010 and 016 in Thailand (Thailand map was generated by QGIS software: a free and open source geographic information system, https://www.qgis.org/en/site/).

**Figure 5:**
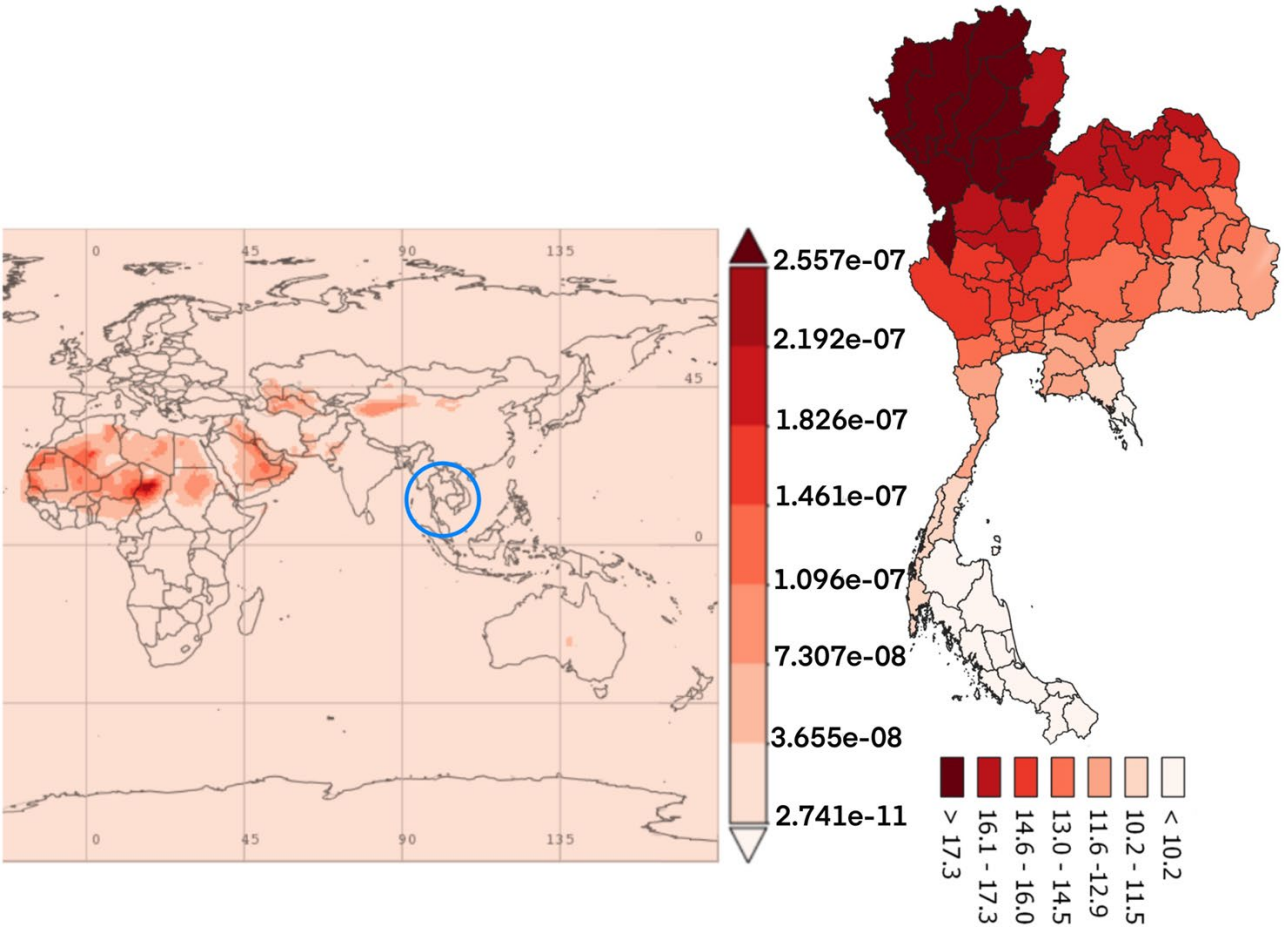
The average time of dust PM_2.5_ concentration monthly over 2010–2016. (The world map was generated by Giovanni Version 4.34: a free and open source earth data, https://giovanni.gsfc.nasa.gov/giovanni/) and accumulated dose between 2010 and 2016 in Thailand (Thailand map was generated by QGIS software: a free and open source geographic information system, https://www.qgis.org/en/site/).

**Figure 6:**
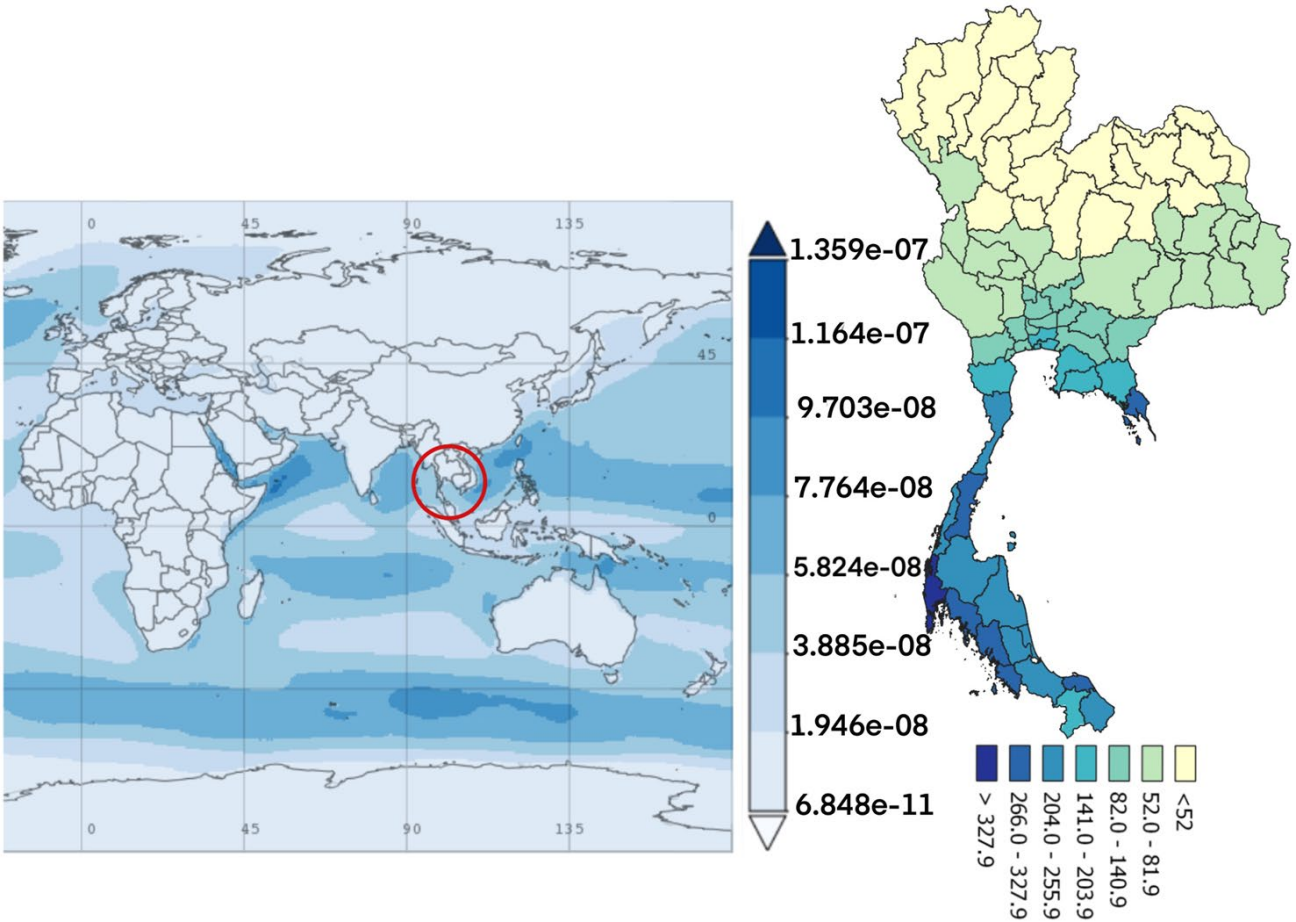
The average time of sea salt concentration monthly over 2010–2016. (The world map was generated by Giovanni Version 4.34: a free and open source earth data, https://giovanni.gsfc.nasa.gov/giovanni/) and accumulated dose between 2010 and 2016 in Thailand (Thailand map was generated by QGIS software: a free and open source geographic information system, https://www.qgis.org/en/site/).

**Figure 7:**
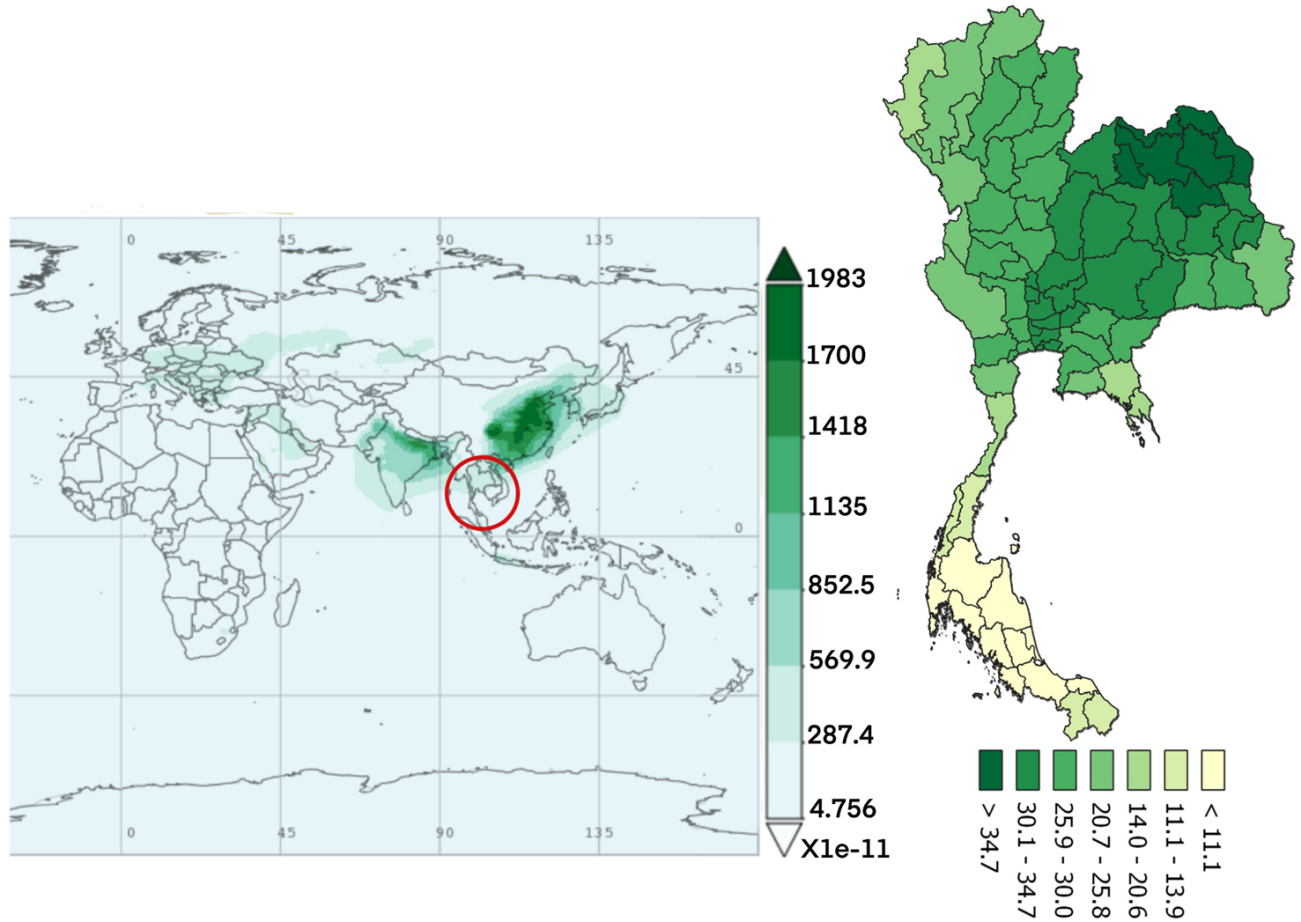
The average time of SO_4_ concentration monthly over 2010–2016. (The world map was generated by Giovanni Version 4.34: a free and open source earth data, https://giovanni.gsfc.nasa.gov/giovanni/) and accumulated dose between 2010 and 2016 in Thailand (Thailand map was generated by QGIS software: a free and open source geographic information system, https://www.qgis.org/en/site/).

- Patient database: the new cases of 59,605 colon cancer patients from various provinces of Thailand according to the database of the Strategy and Planning Division, Ministry of Public Health of Thailand between January 1, 2017 and December 31, 2017 were included. The inclusion criteria were all patients diagnosed based on ICD-10 coding as clinical information of a primary malignant neoplasm that affects the colon; and exclusion criteria was those diagnosed as a malignant carcinoid tumor of the colon. The patients were those who registered for the treatment under the health regions, in which the health regions were divided following geographical location. The patients were recruited from all the regions and then could be represented the characteristics of the patients throughout the country. However, migration of the patients was considered as uncontrollable individual risk and it is one of the limitations of this present study in addition to the patient’s underlying diseases, risks in day-to-day living and risks through the genetics in each family. Table 1 shows estimated incidence of colon cancer specified by provinces and health regions (per 100,000 population).
- Land use: information in regards to the map of Thailand was based on QGIS software for composing and exporting graphical maps^16^. The geographical location of Thailand was identified and displayed the coordinates between latitudes of 5.77434 and 20.43353 and longitudes of 97.96852–105.22908 with a total of 77 provinces. Within the 77 provinces, there are 13 health regions. Thailand’s national health policy oversees and operates most government health facilities while also allocates funding throughout 13 health regions across the country. The Universal Coverage Scheme (UCS) is one of the public health protection schemes. All Thais were covered by health insurance guaranteeing them access to a comprehensive package of health services. This scheme is administered by the National Health Security Office (NHSO) which has 13 regional offices nationwide. The 12 health regions consist of 4–8 provinces with the population of 3–6 million. It aims to provide better quality medical services for citizens within that region. The remaining is the health region 13 which covers the capital city, Bangkok^17^. The map is displayed with the different incidence rate of cancer on each province (Fig. 2).
- Dust pathogen (Black Carbon Surface Mass Concentration or black carbon; Dust Surface Mass Concentration—PM_2.5_ or mineral dust; Organic Carbon Surface Mass Concentration or organic carbon; Sea Salt Surface Mass Concentration or sea-salt; and SO_4_ Surface Mass Concentration or SO_4_): Analysis of dust pathogen was based on data collection gathered and reported by the NASA's satellite bases. Each composition of dust was displayed and analyzed for geophysical parameters in grids from Giovanni's data products and services from the year 2010–2016 displayed the factors that enable cancer in boundary accumulated dose. The entire database was compiled from the database of the aerosol diagnostics model, Modern-Era Retrospective Analysis for Research and Applications, Version 2 (MERRA-2). The filtered MERRA-2 data were included using the mean level of individual grid cells for each month. Dust analytic system represented ambient air quality, including a description of the horizontal and vertical grids between 2010 and 2016. A cubed-sphere grid was the main tool used for computing. The MERRA-2 process and analysis algorithm model had the same characteristics as the recent versions of Gridpoint Statistic Interpolation analysis system (GSI) by controlling variable for humidity. The advantage of MERRA-2 was that it provided detailed data analysis every 3 h and could identify the parameters for observation. The mean value for each of the substances by monthly was utilized and also variables were controlled as an aerosol–climate and aerosol–weather interactions^18^. The mean value of each substance was exported by graphical map and compared with accumulated dose in Thailand (Figs. 3, 4, 5, 6, 7).

**Table 1:** Distribution of incidence rate of colon cancer in 2017 and accumulated aerosol surface mass concentration between 2010 and 2016.

Health region	Incidence rate of colon cancer/100,000 population	Incidence rate of male colon cancer/100,000 population	Incidence rate of female colon cancer/100,000 population	Aerosol surface mass concentration				
				Black carbon	Organic carbon	Sea salt	Dust PM_2.5_	SO_4_
1	104.834	114.108	95.963	7.249	66.436	34.989	18.921	25.35
2	92.242	103.74	81.206	8.161	55.499	53.432	18.151	28.424
3	84.438	91.928	77.35	10.347	57.461	57.147	16.497	28.027
4	82.904	89.33	76.817	11.954	57.786	101.248	14.664	31.738
5	89.594	97.625	82.118	11.464	54.306	129.793	13.99	26.136
6	70.796	75.9	65.965	7.059	44.176	172.054	12.181	25.446
7	91.685	99.557	83.962	9.853	54.221	53.162	15.142	34.498
8	54.426	55.965	52.89	8.926	60.126	40.24	16.598	36.989
9	75.088	72.62	77.525	9.267	51.488	64.186	14.069	31.534
10	71.692	74.158	69.204	6.863	51.442	62.566	13.273	30.272
11	67.564	72.658	62.545	3.443	21.905	306.737	10.681	10.993
12	59.68	60.741	58.63	4.244	23.231	248.977	9.623	10.999
13	169.97	199.14	143.99	10.802	53.281	180.296	13.558	30.674

### Statistical analysis

1. The spatial autocorrelation within the spatial data was used to define the relationship between time of air quality—place in accumulated dose and the burden of the disease. In addition, all of the data were also used to analyze the density of substances in each cell grid area. With the health aspect of air pollution reported from the WHO European Centre for Environment that sea salt is not classified as a hazardous compound and it is plausible that at current exposure levels no harmful effects will occur with certainty in humans^19^. Possible interactions of various substances and the air quality profile from MERRA-2 was analyzed by the individual risk, except for the sea salt, and the incidence rate ratio of colon cancer were estimated using the Poisson log-linear model. All of the parameters such as dust-PM_2.5_, organic carbon, black carbon, sea salt, and SO_4_ were adjusted in the accumulated dose from 2010 to 2016. This subsequent procedure was compared the incidence rate ratio by each health region. The low incidence rate area was used as comparable base with other regions for estimation of the high-risk zone. A systematic component of the model demonstrated as:
  $${\log}\left( \mu \right) = \alpha + \beta {\text{x or equivalently}},\quad \mu = {\exp}\left( {\alpha + \beta {\text{x}}} \right) = {\exp}\left( \alpha \right){\exp}\left( {\beta {\text{x}}} \right)$$
  which can easily show that: exp(α) is the effect on the mean of Y, when X = 0, which is μ. exp(β) is the corresponding predictor variable has multiplicative effect of exp(β) on the mean of Y per unit increase in X, which is μ.
  A consequence of the above is that:
  (i) In case β = 0, then exp(β) = 1, and the expected value, μ = E(y) = exp(α), and Y and X are clearly not correlated.
  (ii) In case β > 0, then exp(β) > 1, and the expected value μ = E(y) is exp(β) times larger than when X = 0.
  (iii) In case β < 0, then exp(β) < 1, and the expected value μ = E(y) is exp(β) times smaller than when X = 0.
2. Linear predictor of colon cancer incidence rates ratio by substance at the health region were analyzed by the integrated nested Laplace approximation (INLA) approach. The INLA method is explicated in the developing model of the INLA for approximate Bayesian inference. This process acts as an alternative to the traditional Markov chain Monte Carlo methods. The INLA pattern calculates the comparison between the posterior marginals and hyperparameters. This has been accomplished by utilizing the mathematical characteristic of GMRF and Laplace approximation for multidimensional integration. An accessible method for the best fit model was implemented for hierarchical models with a summary of the posterior distribution of the intercept boundary analysis for the real data. The INLA method was used to approximate the spatial fields and detect which substance produced the risk factors that correlated with cancer cases throughout the year by integrating explicative variables such as the mean household income. This investigation was able to identify and quantify the model which estimates the outcome and measures the assessment of convergence, sensitivity analyses performed, relative risk and the determination of significant geographical variations.

The integrated nested Laplace approximation was used in order to fit models into spatial data in a Bayesian context.

$$\eta = \alpha + \sum {\text{nfj}} = 1{\text{f}}\left( {\text{j}} \right)\left( {{\text{uij}}} \right) + \sum {\text{n}}\beta {\text{k}} = 1\beta {\text{kzki}} + \varepsilon {\text{i}}\eta = \alpha + \sum {\text{j}} = 1{\text{nff}}\left( {\text{j}} \right)\left( {{\text{uij}}} \right) + \sum {\text{k}} = 1{\text{n}}\beta \beta {\text{kzki}} + \varepsilon {\text{i}}$$

η is the linear predictor for a generalized linear model formula, u is a linear function of some variables, β is the effects of covariates, z and ϵ are an unstructured residual.

## Results

The dust particles in the atmosphere are characterized by suspension of solids and liquids particles in the air. Aerosol particles in this research included precipitation, aerosol, black carbon, dust, organic carbon, sea salt, and SO_4_ demonstrated each year from the database of the MERRA-2 in aerosol diagnostics model. For the results, the distribution of accumulated air pollution intensity in Thailand between 2010 and 2016 is displayed in Table 1. It showed the highest incidence of colon cancer in the health region 13 with 169.970/100,000 population which represented the capital city, Bangkok and 54.426/100,000 population, the lowest incidence rate in the health region 8. Cancer incidence rates are higher among males than females. Figure 2 also demonstrates the distribution of diseases by spatiotemporal distribution as a province scale and the health region. Map A shows five provinces with extremely dense with high incidence rate of colon cancer (dark red color) and ten provinces for low incidence rate (light color). Map B represents the health regions, showing the incidence rates were clustered in the northern and the central part of Thailand, especially in the health region 13, 1 and 2. Time averaged world map of the individual substance such as black carbon, dust, organic carbon, sea salt, and SO_4_ concentration monthly over 2010–2016 and accumulated dose between 2010 and 2016 in Thailand (Thailand mapping) are displayed in Figs. 3, 4, 5, 6 and 7.

One of the procedures used for assessing spatio-temporal ecological model was a prerequisite for addressing the Poisson generalized linear model. We compared the simulation models of the inverse-variance method of both univariate and multivariate analysis and estimated IRR of colon cancer using the lowest incidence rate category (the health region 8) as reference. This health region is composed of seven provinces around the North-Eastern region; Loei, Nong Bua Lam Phu, Udon Thani, Nong Khai, Bueng Kan, Sakon Nakhon, and Nakhon Phanom. We applied these methods to weight the study-specific IRRs to generate a pooled estimate and compared of baseline variability and heterogeneity in the intervention effect. The following distributions had been taken into consideration with 95% credible intervals. The risk-exceedance probabilities are shown in Table 2. Results, therefore, indicated that the occurrence of incidence rates of colon cancer when compared to baseline is greatest in the health region 13. With a significant influence on increasing of 1 μg/m^3^ black carbon, organic carbon, Dust-PM_2.5_, and SO_4_ at this health region were associated with increased cancer risk with 2.495, 2.704, 2.383, and 2.676 times, respectively. Other zones were indicated some significantly different value in risk.

**Table 2:** Adjusted incidence rate ratio of colon cancer by the health regions.

Health region^a^	IRR	95% CI	P value	IRR	95% CI	P value
	Black carbon			Organic carbon		
1	1.814	1.739–1.891	< 0.001	1.650	1.580–1.722	< 0.001
2	1.411	1.345–1.480	< 0.001	1.446	1.377–1.518	< 0.001
3	1.286	1.221–1.353	< 0.001	1.345	1.278–1.416	< 0.001
4	1.038	0.987–1.091	0.147	1.127	1.077–1.180	< 0.001
5	1.342	1.282–1.406	< 0.001	1.478	1.414–1.545	< 0.001
6	1.204	1.151–1.259	< 0.001	1.316	1.249–1.387	< 0.001
7	1.484	1.421–1.550	< 0.001	1.585	1.516–1.657	< 0.001
9	1.261	1.209–1.315	< 0.001	1.362	1.245–1.426	< 0.001
10	1.294	1.233–1.359	< 0.001	1.307	1.502–1.372	< 0.001
11	1.351	1.276–1.431	< 0.001	1.643	1.334–1.796	< 0.001
12	1.192	1.128–1.259	< 0.001	1.456	1.722–1.590	< 0.001
13	2.495	2.397–2.597	< 0.001	2.704	2.630–2.853	< 0.001

Health region^a^	IRR	95% CI	P value	IRR	95% CI	P value
	Dust PM_2.5_			SO_4_		
1	1.872	1.783–1.966	< 0.001	1.851	1.739–1.970	< 0.001
2	1.446	1.375–1.521	< 0.001	1.446	1.365–1.531	< 0.001
3	1.320	1.254–1.389	< 0.001	1.375	1.292–1.463	< 0.001
4	1.056	1.004–1.111	0.035	1.145	1.089–1.204	< 0.001
5	1.329	1.263–1.398	< 0.001	1.496	1.408–1.589	< 0.001
6	1.040	0.976–1.109	0.227	1.227	1.156–1.303	< 0.001
7	1.457	1.392–1.525	< 0.001	1.533	1.466–1.602	< 0.001
9	1.180	1.121–1.241	< 0.001	1.301	1.241–1.364	< 0.001
10	1.101	1.033–1.173	0.003	1.278	1.207–1.353	< 0.001
11	1.005	0.923–1.094	0.909	1.362	1.214–1.527	< 0.001
12	0.881	0.803–0.966	0.007	1.218	1.087–1.364	0.001
13	2.383	2.264–2.509	< 0.001	2.676	2.556–2.802	< 0.001

*IRR* incidence rate ratio, *CI* confidence interval.

^a^Reference: health region-specific incidence rate ratio are all expressed in relation to the lowest incidence rate ratio area (the health region 8).

**Figure 8:**
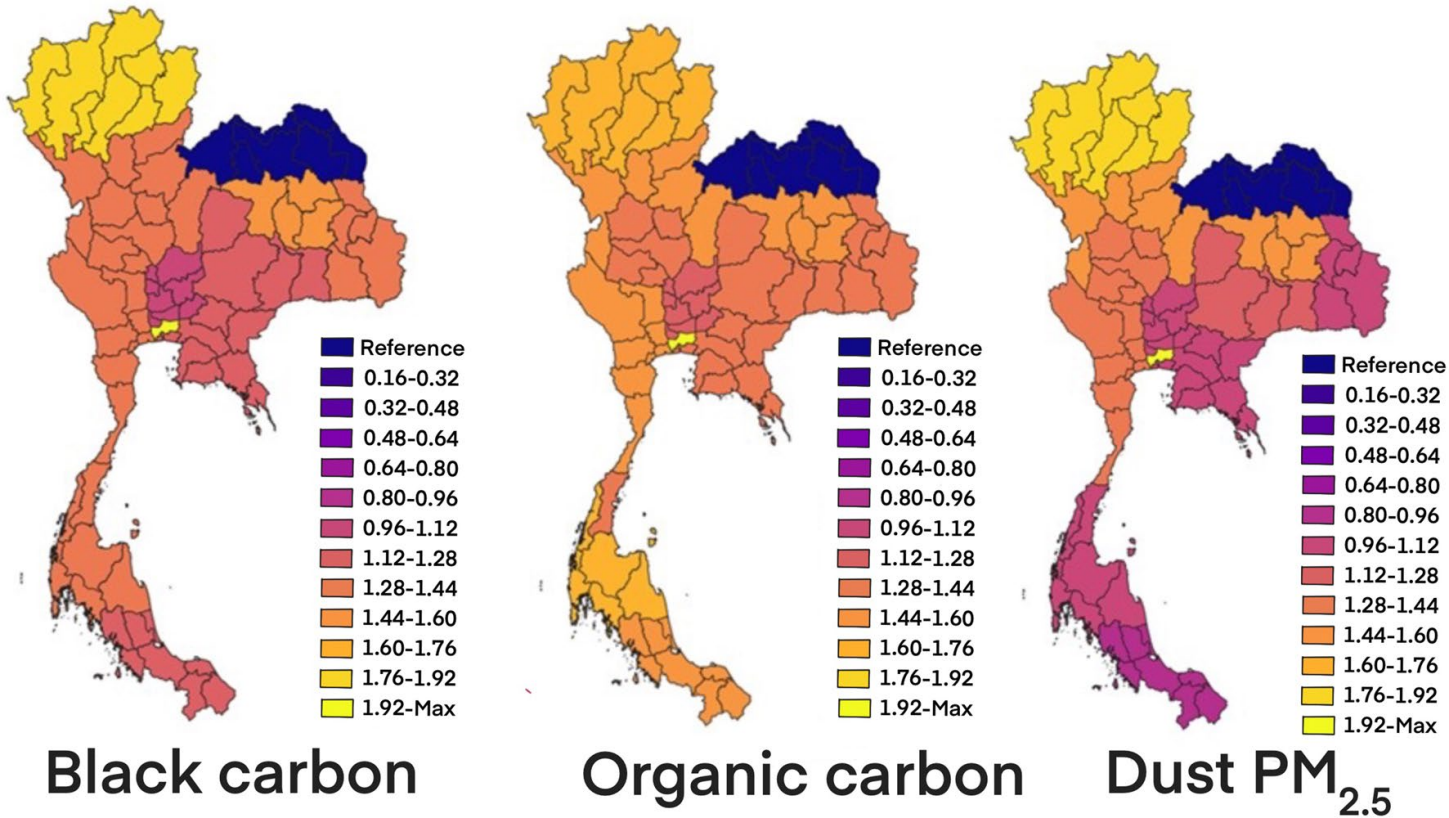
Ecological regression: posterior mean value for black carbon, organic carbon and dust-PM_2.5_ for IRR of colon cancer at the health region level in 2017. (The maps were generated by QGIS software: a free and open source geographic information system. https://www.qgis.org/en/site/).

The multivariate analysis (Table 3) revealed possible spatial patterning of the posterior probabilities of risk and this pattern allowed us to qualitatively assess commonalities among cities and identify the potential signal of aerosol substances-related colon cancer incidence. For example, the posterior probabilities of risk appeared the most in dust PM_2.5_. It could be interpreted as relative risk in every increase of 10 μg/m^3^ in black carbon, organic carbon, and dust-PM_2.5_ levels were associated respectively with an increase of 4%, 4%, and 15% in the risks of colon cancer. The distribution throughout the country of the aerosol substances-related colon cancer incidence is shown in Fig. 8. Health region-specific IRR of colon cancer which reported for a difference in the lowest incidence rate zone- that is, the adjusted rate ratios across the range of exposure for each pollutant among the 13 health regions. For the effect of difference of Poisson regression, rate ratios were estimated for black carbon, organic carbon and dust-PM_2.5_ levels. Hence the relative risk of colon cancer at the health region 13 compared to the health region 8 was more than 1.92 times.

**Table 3:** Posterior marginals by multivariate analysis for linear predictor incidence rates ratio and computed fitted values of colon cancer.

Fixed effects	Mean	SD	2.50%	50%	97.50%
Black carbon (10 μg/m^3^)	0.04	0.02	0.01	0.04	0.07
Organic carbon (10 μg/m^3^)	0.04	0.00	0.04	0.04	0.05
Dust PM_2.5_ (10 μg/m^3^)	0.15	0.02	0.12	0.15	0.19
SO_4_ (10 μg/m^3^)	0.00	0.01	0.01	0.00	0.01

*SD* standard deviation.

## Discussion

Air pollution can cause a variety of adverse health effects. Airborne particulate matter (PM) is a major factor regarding health issue, especially cancer chances^14^. PM chemical composition was mainly with organic carbon, inorganic carbonaceous material or black carbon or elemental carbon, elemental dust or crust-derived minerals, sea salt, and sulfate^19,20^. Organic carbon was a material of biological origin whereas black carbon was a material produced by the incomplete combustion of heavy petroleum products such as coal tar or diesel emissions^21^. The elements components in PM possibly derive from topsoil erosion and dust resuspension distributed in the coarse mode of dust PM^22^. The characteristics of metal concentration in PM differ from place to place as well as from size to size^23^.

The development of malignancy tumors are complex multistep processes attributed by many risks. It is widely known that dust particles of such pollution are related to cancer of many organs^24,25^. The pollutant elemental dust composed of the concentrations of heavy metals such as lead, zinc, nickel, copper, cadmium and chromium^26^. One of the most common places associated with potentially toxic metal in local environment was the street dusts(SDs)^27^. There are many routes of entry through body, namely inhalation, food contamination and skin contact^28^. Cadmium was a heavy metal contaminant whose toxicity was associated with colorectal cancer. It induced the carcinogenic effects, following by the reactive oxygen species (ROS) pathways^29^. The tissue sampling, compared between cancerous and non- cancerous lesion, showed the significant higher levels of polluted metals in pathological one^30^. Even in higher blood levels of cadmium and lead, these may promote the occurrence and progression of gastrointestinal cancers^31^.

Black carbon is produced by incomplete combustion of carbon-containing materials. The organic compounds or polycyclic aromatic hydrocarbons (PAHs) can be extracted from particle surfaces of black carbon^32^. This also explained with carcinogenic effects of soot. An important substance in the PAHs is benzo [a] pyrene, which is a carcinogen^33,34^. PAHs dissolves well in fat, therefore they can be absorbed into the body through the skin, eating and breathing. PAHs’ absorption into the gastrointestinal tract occurs when combined with bile that helps to absorb through the intestines, but the process of eliminating these toxins occurs when peeling the epithelium and the excretion of mucus in the colon^35^. When PAHs enters the body, they are metabolized by cytochrome P450, which depends on the type of PAHs. The key carcinogen is benzo (a) pyrene, which is benzo [a] pyrene-7, 8-dihydrodiol-9, 10-oxide (BPDE) or benzo[a] pyrene diol epoxide is metabolized. This metabolized has high sensitivity to catch with DNA. When DNA is replicated and fragmented, which abnormal matching base substances occurred, resulting in a mutation^36^. The experimental models provided the potential cellular mechanisms of black carbon initiate to oxidative stress, DNA methylation and formation of DNA adducts^21^.

Organic carbon emitted from vegetation or wood smoke. It is unusual to differentiate the effect of organic carbon from black carbon. Most of the studies demonstrated the health effects with the organic compound and black carbon^37^. Only the few studies showed the association of lung cancer and cardiovascular disease but did not clarify with the exactly mechanism^14,38^. Further work, therefore, is needed to confirm these findings.

With the time specific risk of exposure with the carcinogen, only the evidence of long term exposure to the toxic substance is able to accelerate the colon carcinogenesis process but the exact time-risk involved has not been evident^39^. There were few evidences that showed more than 5 years exposure to air pollution increased mortality from respiratory disease, lung cancer and cardiovascular disease^14,40^. This research used NASA’s (National Aeronautics and Space Administration) satellite data to measure the quality of aerosol substances that demonstrated ambient air quality then compared the incidence of colon cancer by demographics (province and health region). In other studies, polluted substances are detected by satellite, in which those correlated with mortality rates were PM_2.5_, nitrogen dioxide (NO_2_) and ozone (O_3_)^41^ but not correlated with the gastrointestinal tract diseases. A trial tested more than 5 years effects of air pollution in colon cancer has been developed. With the several limitations of the inability to corporate individual specific health status information, it made unadjusted effects for diet and lifestyle, or even population dynamics of migration which might be interfered by the analysis results. This present study reveals that accumulated ambient air quality that was detected with the aerosol diagnostics model by MERRA-2, was associated with an increased risk of colon cancer, especially in dust-PM_2.5_, black carbon and organic carbon. Sulfate, however, was not significant for multivariate analysis and it seems like having more influence with the respiratory tract pathology^14,42^. Although many studies have shown that colon cancer is caused partly by environmental conditions, there were no reports of colon cancer and the model of aerosol diagnostics by the NASA’s satellite data prior. To our knowledge, this is the first study to investigate the relevance of specific ambient air substances quality and colon cancer using advanced technological screening.

## Conclusion

In everyday life, people are exposed to many toxic substances that may or may not associate with cancer. However, being exposed to high levels of carcinogens is a serious risk to life. Consistent with this present study, a significant increase in the incidence of colon cancer with ambient air quality is statistically significant and lead concerns regarding the prevention of air pollution. This study uses the nation’s database on the incidence of colon cancer, which is able to link to the pollution database of NASA technology which has not been researched in Thailand before. This process can be monitored and covered all the provinces of Thailand.

## Data Availability

The datasets used and/or analyzed in the present study can be obtained from the corresponding author on reasonable request.
